# Parasitism-Induced Changes in Microbial Eukaryotes of Peruvian Alpaca Gastrointestinal Tract

**DOI:** 10.3390/life14020187

**Published:** 2024-01-27

**Authors:** Diana Sanchez, Celso Zapata, Yolanda Romero, Nils H. Flores-Huarco, Oscar Oros, Wigoberto Alvarado, Carlos Quilcate, Hada M. Guevara-Alvarado, Richard Estrada, Pedro Coila

**Affiliations:** 1 Unidad de Post Grado de la Facultad de Medicina Veterinaria y Zootecnia, Universidad Nacional del Altiplano de Puno, P.O. Box 291, Puno 21001, Peru; dsanchez@epg.unap.edu.pe (D.S.); dbionils@gmail.com (N.H.F.-H.); 2 Facultad de Medicina Veterinaria y Zootecnia, Universidad Nacional del Altiplano de Puno, P.O. Box 291, Puno 21001, Peru; czapata@unap.edu.pe (C.Z.); odoros@unap.edu.pe (O.O.); 3 Instituto de Investigación en Bioinformática y Bioestadistica (BIOINFO), Av. Raúl Ferrero 21, Lima 15024, Peru; yolanda888@hotmail.com; 4 Dirección de Desarrollo Tecnológico Agrario, Instituto Nacional de Innovación Agraria (INIA), Av. La Molina 1981, Lima 15024, Peru; promegnacional@inia.gob.pe; 5 Facultad de Ingeniería Zootecnista, Agronegocios y Biotecnología, Universidad Nacional Toribio Rodríguez de Mendoza de Amazonas (UNTRM), Cl. Higos Urco 342, Chachapoyas 01001, Peru; wigoberto.alvarado@untrm.edu.pe (W.A.); hada.guevara@untrm.edu.pe (H.M.G.-A.)

**Keywords:** alpacas, metabarcoding, microbiota diversity, parasites, biomarkers, NGS

## Abstract

Alpacas, important genetic resources in the Andean region of Peru, are vulnerable to diarrhea caused by pathogenic parasites such as *Eimeria lamae* and *Giardia* sp., which can be fatal, especially in neonates, due to their physiological immaturity and limited adaptability. The study investigated the diversity and abundance of intestinal fungi and protists in alpacas infected with *Eimeria lamae* and *Giardia* sp. compared to healthy alpacas. A total of 19 alpacas, aged between one and two months, were included. They were divided into two groups, one with pathological conditions (nine) and the other healthy (ten). Parasitological analyses for the detection of parasites and subsequent molecular analysis were performed on the collected fecal samples. The results revealed a greater diversity and abundance of protists in infected alpacas in comparison with healthy alpacas, while the fungal composition did not show significant changes. Therefore, parasitic infections affect the protist component of the alpaca gut microbiota. Also, it was observed that *Blastocystis* was identified in all healthy alpacas, serving as a possible marker of the health of the intestinal microbiota; in addition, *Prussia* and *Pichia* are beneficial fungi that help control diseases. This groundbreaking study in neonatal alpacas is the first to explore potential changes in the intestinal microbiota during an infectious state, underscoring the importance of further research to comprehend its effects on alpaca health and immune responses.

## 1. Introduction

In the Peruvian Andes, alpacas stand as indigenous domestic camelids crucial to the region’s genetic diversity [1]. These animals, predominantly of the Huacaya and Suri breeds, contribute significantly to the high-altitude areas of the Andes, with Huacaya prized for the quality of its fiber [2,3,4]. However, diarrhea in alpacas poses a severe threat, particularly to newborns (crias), and is often the leading cause of mortality [5]. This condition results in nutrient and water loss, leading to energy deficits, weakness, and increased mortality, especially among crias due to their physiological immaturity [6,7].

The most common infectious pathogens responsible for diarrhea in alpacas include *Salmonella* sp., *Escherichia coli*, *Giardia* sp., *Coccidia*, and *Cryptosporidium* sp. [8]. The vulnerability to gastrointestinal pathogens emphasizes the significance of comprehending and addressing diarrhea in alpacas, particularly among the susceptible cria population. In this context, the diagnosis of coccidiosis often occurs in neonates and juvenile camelids [9]. Oocysts cause direct damage to the epithelial mucosa of the small intestine, resulting in the development of enteritis and diarrhea [10]. The etiology of coccidiosis, an enteric parasitic disease, manifests through symptoms such as anorexia, the onset of colic, diarrhea, and, eventually, sudden death, especially in neonates [11]. Moreover, five species of *Eimeria* have previously been identified as causative agents of diseases: *E. ivitaensis*, *E. lamae*, *E. macusaniensis*, and *E. punoensis* [12,13].

Giardiasis is a prevalent disease in ruminants, manifesting with symptoms like diarrhea, weight loss, and malabsorption. However, asymptomatic infections are also frequently observed. The etiology of giardiasis involves fecal–oral transmission through direct contact with infected humans or animals and the ingestion of water or food contaminated with cysts, highlighting the importance of proper hygiene and sanitation practices in its spread [14]. *Giardia* was initially reported in llamas in 1987 [15]. However, there has been a limited amount of research concerning the presence of *Giardia* in alpacas [16,17,18,19].

Intestinal parasites, particularly protozoa, can disrupt the structure of the intestinal microbiota, leading to diseases [20]. Host defense strategies against parasitic infections, including immune responses, are triggered in response to parasite invasion [21]. However, the immune system plays a crucial role in regulating the intestinal microbiota and its relative composition to maintain a mutually beneficial symbiotic relationship between the host and microorganisms [22]. Species belonging to the *Eimeria* genus influence host immune responses to facilitate their invasion and colonization by reducing the production of inflammatory cytokines [22], thus altering the balance of the intestinal microbiota. Nevertheless, there are studies suggesting that *Giardia* may protect against the development of diarrheal diseases by modulating the immune response, although further research is needed in this regard [23].

Metagenomics plays a pivotal role in scientific exploration, offering intricate insights into microbial communities within diverse environments, including the intestinal tract [24]. The use of the 18S rRNA gene in biodiversity and ecology studies is notable for its ubiquity in eukaryotic organisms and its divergence in different loci. This versatile molecular tool allows for the acquisition of insightful data on the taxonomy and phylogeny of microbial communities, making a significant contribution to our understanding of ecosystem structure and function [25].

Although the gut microbiota plays a significant role in ruminants [26], information about the microbial community in alpacas is limited, especially beyond the stomach, and a proper understanding is lacking about how this community responds to dietary imbalances [27]. The main objective of this study is to acquire a greater understanding of the composition of the microbial community in alpacas and how it is influenced in response to episodes of diarrhea, taking into account the presence of two pathogens, *Eimeria lamae* and *Giardia* sp. Such research will help further our understanding of the complex interactions between gut microbiota, dietary factors, and health outcomes in alpacas.

## 2. Materials and Methods

### 2.1. Animal and Sample Collection

A total of nineteen alpacas, aged between one and two months, were obtained from the La Raya Experimental Center, an entity belonging to the National University of the Altiplano Puno. The selection of these specimens was carried out in compliance with the Peruvian National Law No. 30407, relating to ‘Animal Protection and Welfare’.

The sample consisted of 9 alpacas with pathological conditions and 10 healthy alpacas, with an equal distribution of males and females in the group with diarrhea (4 males and 5 females). The pathological conditions in the affected alpacas were diagnosed by veterinarians affiliated with the Animal Health Laboratory of the Professional School of Veterinary Medicine at the National University of San Antonio Abad del Cusco. All individuals received a diet designed to meet their particular nutritional needs as alpacas. Hemoglobin levels, leukocyte counts, heart rate, and respiratory rate were assessed in alpacas overall.

To maintain sample integrity, we separated the alpacas with diarrhea from the healthy ones a day before sampling, using sterile tools under aseptic conditions. The collected samples were transferred to sterile 50 mL plastic containers and quickly transported to the laboratory, where they were initially stored at 5 °C for subsequent parasitological analysis and subsequently at −80 °C for subsequent analysis.

### 2.2. Parasitological Study of Feces 

A direct microscopic examination was performed, using parasitological lugol as a stain [28], for the identification of protozoan cysts (*Giardia* sp.) and oocysts (*Eimeria lamae*) in fecal samples from affected alpacas. Then, qualitative flotation concentration methods were applied with saturated sucrose solution [29], followed by the implementation of the modified McMaster quantitative method [30], specifically in fecal samples from alpacas with pathological conditions.

### 2.3. DNA Extraction and Sequencing 

Genomic DNA was extracted from fecal samples using the PureLink microbiome DNA purification kit (Invitrogen, Waltham, MA, USA). To assess DNA quality, Qubit^®^ was used for quantification, and DNA integrity was visualized by running an electrophoretic gel on 1% agarose. This DNA was prepared for V4 sequencing using primer 528 forward, 5′-GCGGTAATTCCAGCTCCAA-3′ and 706 reverse, 5′-AATCCRAGAATTTCACCTCT-3′ targeting the 18S rRNA gene [31]. The PCR protocol commenced with an initial denaturation step at 94 °C for 2 min, followed by an initial set of 5 cycles involving denaturation at 94 °C for 45 s, annealing at 52/54 °C for 45 s each, and elongation at 72 °C for 1 min. This was succeeded by 35 additional cycles with a reduced annealing temperature set at 50/52 °C. The process concluded with a final elongation step at 72 °C for 10 min. Subsequently, this region was individually amplified from each sample using the TruSeq^®^ DNA sample preparation kit without PCR from Illumina, along with the appropriate primers. Library quality was evaluated using the Qubit 2.0 fluorometer from Invitrogen and the fragment analyzer from the Agilent Bioanalyzer 2100 system. Amplicon libraries were then sequenced using the 2 × 250 paired-end protocol on the Illumina Novaseq 6000 platform (San Diego, CA, USA).

### 2.4. Bioinformatics Analysis

During the preparation and analysis of the sequencing data, we utilized the Quantitative Insights Into Microbial Ecology (QIIME) analytical platform [32]. Processed through the DADA2 v.1.18 protocol [33], paired-end fastq files underwent handling, and amplicon sequence variants (ASVs) were generated. In the initial stages of the process, quality assessment, trimming, and noise reduction were carried out on the forward and reverse sequences, before their integration into the Amplicon Sequence Variants (ASV), with the purpose of mitigating the potential for inaccurate ASVs. Exclusion was performed for unique sequences whose total abundance did not exceed 10 reads in the global set of samples. The taxonomic categorization of the sequences was carried out by using the naive Bayes classifier integrated into QIIME2, previously calibrated with the Silva Reference v.138.1 database, for the identification of protists and fungi. The alignment of high-quality sequences was carried out using the MAFFT tool [34].

### 2.5. Statistical Analysis

Rarefaction curves were generated for each sample to assess the sequencing depth. Alpha diversity of intestinal fungal and protozoan communities was determined based on the relative abundance distribution of Operational Taxonomic Units (OTUs) in each sample. Statistical analysis of the data was performed using the R package Phyloseq [35] in R (v4.1.1) [36], calculating alpha diversity metrics, such as Observe, Pielou, Shannon, and Simpson, with the MicrobiotaProcess library [37]. Kruskal–Wallis tests were used to evaluate the distribution of these metrics among the analyzed groups. Beta diversity was determined using the Bray–Curtis method and visualized through Principal Coordinate Analysis (PCoA). Differences in fungal and protist communities between groups were evaluated by PERMANOVA analysis with 9999 permutations [38], using the R Vegan package [39], considering values of *p* < 0.05 statistically significant. Additionally, LDA scores were obtained using the LEfSe algorithm.

## 3. Results

In this study, the presence of *Eimeria lamae* oocysts was observed in fecal samples from two-month-old alpacas, while the presence of *Giardia* sp. cysts was detected in samples from one-month-old alpacas. In contrast, no parasites were detected in the control group of alpacas at 1 month and 2 months of age. Regarding two-month-old *Eimeria lamae* samples, an average of 85,750 oocysts per gram of feces (OPG) was observed in samples with diarrhea, whereas no oocysts were found in samples without diarrhea. Similarly, no oocysts were detected in one-month-old alpacas (Table S1). In addition, biochemical data from alpacas in relation to their health status were obtained (Table S2). 

For protists, a total of 3,901,824 high-quality reads were obtained, with an average of 52,577 high-quality reads, a maximum of 1,112,183 high-quality reads, and a minimum of 9032 high-quality reads. Similarly, for fungi, a total of 6,733,314 high-quality reads were generated, with an average of 283,817 high-quality reads, a maximum of 1,219,543 high-quality reads, and a minimum of 34,082 high-quality reads.

### 3.1. Alpha Diversity of the Gut Microbiota in the Alpaca Population

To further explore the variations in the intestinal microbiota communities of fungi (Figure S1) and protists (Figure S2) concerning infection by parasite type, the use of the rarefaction curve was sought. An expected diversity was obtained in the sampling of fungal communities (Figure S1) and protists (Figure S2), this curve demonstrated a preference for optimization. Consequently, the dataset was considered suitable for further analysis.

**Figure 1:**
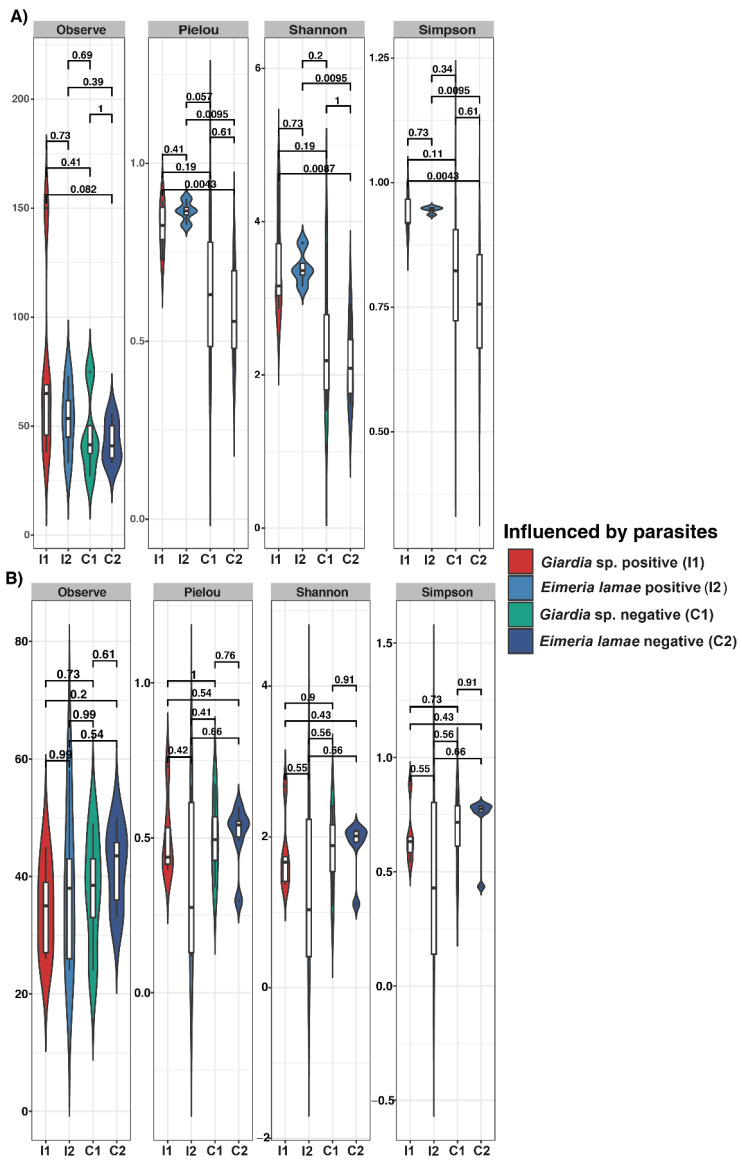
Alpha diversity (Observe, Pielou, Shannon, and Simpson) gut microbiota indices between health conditions with Illness *Giardia* sp., Illness *Eimeria lamae*, *Giardia* sp. negative and *Eimeria lamae* negative. (**A**) Protist alpha diversity gut microbiota index. (**B**) Gut microbiota index of fungal alpha diversity.

The results of the alpha diversity index are shown in Figure 1. For protists (Figure 1A), significant differences were identified between the group of patients diagnosed with *Giardia* sp. at one month and the *Eimeria lamae* negative group, Pielou index (*p* = 0.0087), Shannon index (*p* = 0.0043), and Simpson index (*p* = 0.0043). Significant differences were also observed in the two-month patient group diagnosed with *Eimeria lamae* and the *Eimeria lamae* negative group, Pielou index (*p* = 0.0043), Shannon index (*p* = 0.0043), and Simpson index (*p* = 0.0043). The Shannon and Simpson indices provide a tool to evaluate the diversity present in the intestinal microbiota. The Pielou index reflects the uniformity in the distribution of species abundance in the microbial community, which is crucial to understanding the evenness in the presence of different species in the ecosystem. Greater richness is observed in the groups of patients infected by *Giardia* sp. and *Eimeira lamae* However, in the context of fungal composition (Figure 1B), no statistically significant disparities in alpha diversity were discerned.

### 3.2. Beta Diversity of the Gut Microbiota in the Alpaca Population

**Figure 2:**
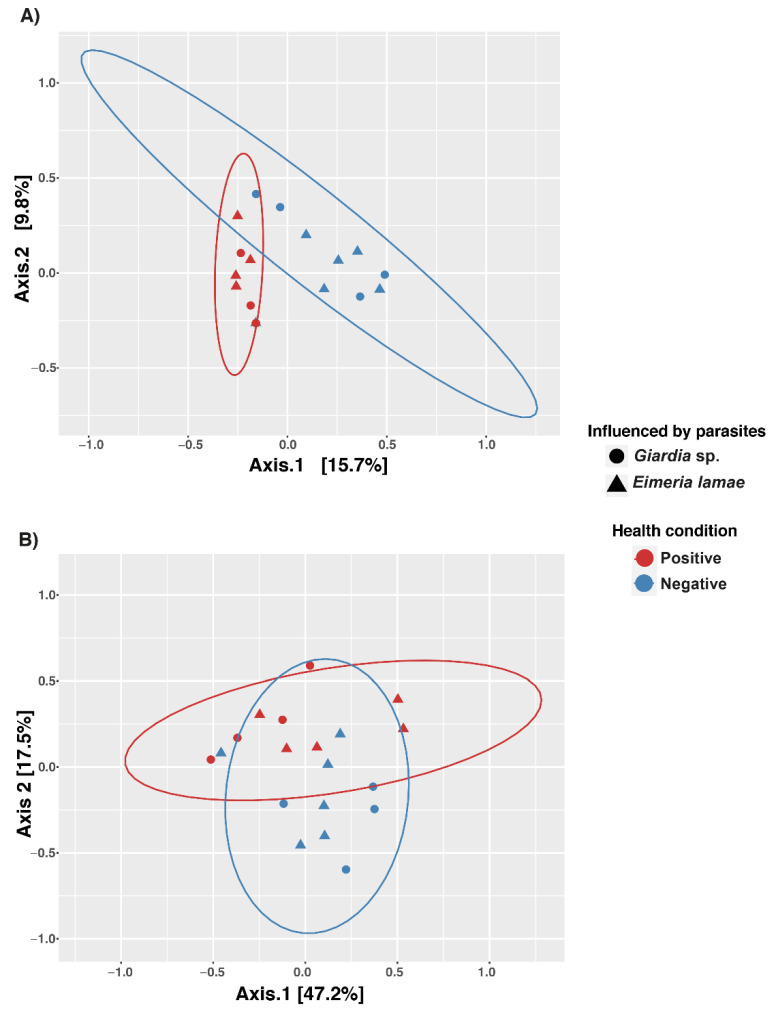
Principal Coordinate Analysis (PCoA) plot of beta diversity based on Bray–Curtis distance derived from sequencing data. The samples are represented by colors and divided into two: healthy (blue) and illness (red) groups. There is also a shape representation for infection by *Giardia* sp. (circle) and *Eimeria lamae* (triangle). (**A**) Beta diversity of protists. (**B**) Beta diversity of fungi.

Principal Coordinate Analysis (PCoA) was conducted (Figure 2). When comparing the similarity of protist composition (Figure 2A), a notable convergence was observed in both healthy and diseased groups concerning the influence of parasites such as *Eimeria lamae* and *Giardia* sp., resulting in wider distance dispersion. The Adonis test further validated this observation, a statistical analysis highlighting the influence of health status. Health status demonstrated statistical significance with a *p*-value of 0.0003 (Table 1), further emphasizing the influential role of health status in the composition of intestinal protist microbiota.

Similarly, the analysis was conducted for the fungal group (Figure 2B), revealing a slight separation in the dispersion of groups based on health status. This finding was corroborated by the Adonis test, yielding a significant *p*-value of 0.0092 (Table 1), thus emphasizing the influence of health status on the composition of the intestinal fungal microbiota.

### 3.3. Correlation between Biochemical Parameters and the Alpha Diversity of Protozoan Composition in the Microbiota

**Figure 3:**
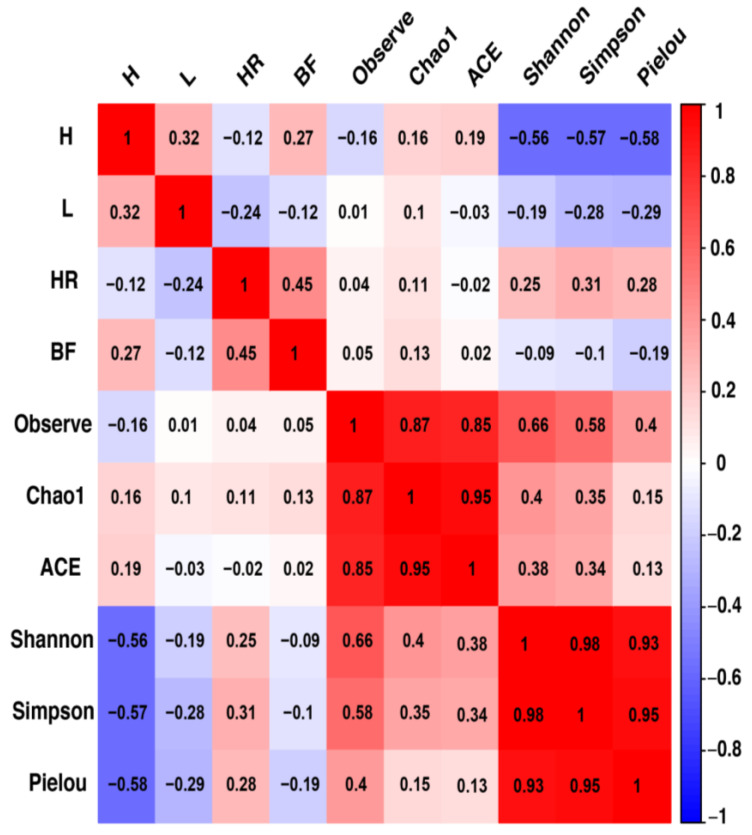
Correlation matrix between biochemical parameters and alpha diversity indices of protists. Positive correlations are depicted in red, while negative correlations are shown in blue. The color intensity and dot size are proportional to the correlation values within each correlation group. H: Hemoglobin; L: Leukocyte; HR: Heart Rate; BF: Respiratory Rate. Alpha diversity indices: Observed, Chao1, ACE, Shannon, Simpson, and Pielou.

Correlations between biochemical parameters and protist alpha diversity indices were evaluated in this study, as presented in Figure 3. A significant negative correlation was observed between hemoglobin levels and alpha diversity profiles, specifically Shannon, Simpson, and Pielou indices, with statistical significance of *p* = 0.0126, *p* = 0.0116, and *p* = 0.00983, respectively.

### 3.4. Composition Taxonomic of the Gut Microbiota

**Figure 4:**
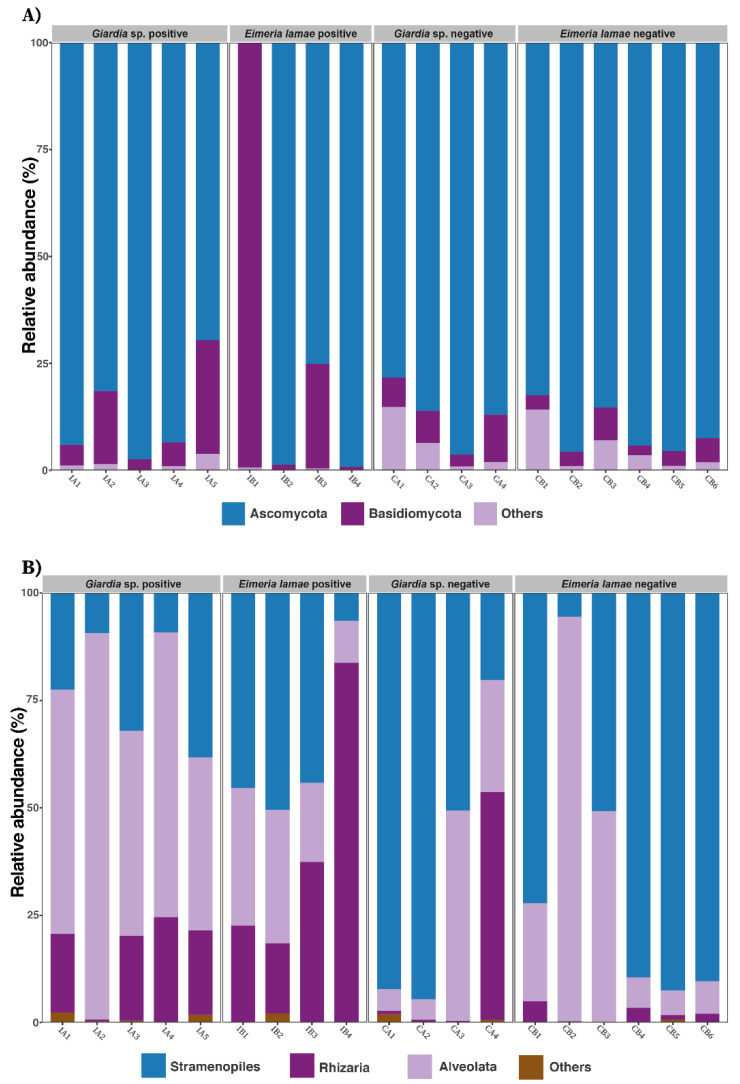
Relative abundances were observed in 1-month disease groups infected with *Giardia* sp. and 2-month illness groups infected with *Eimeria lamae*, in comparison with *Giardia* sp. negative and *Eimeria lamae* negative groups. Only the most represented taxa are presented. (**A**) Relative abundance of the most predominant phyla in fungi. (**B**) Relative abundance of the most abundant classes in protists.

Figure 4 presents the composition at the phylum level in fungi and at the class level in protists. The results indicate that concerning fungi (Figure 4A), in the intestinal microbiota of the Illness *Giardia* sp., Illness *Eimeria lamae*, *Giardia* sp. negative and *Eimeria lamae* negative groups, the phyla Ascomycota (25%, 21.88%, 20%, and 31.94%) and Basidiomycota (41.44%, 19.25%, 20.17%, and 18.90%) were the predominant phyla, representing approximately 97% of the total fungal composition.

Regarding class abundance in protists (Figure 4B), it was observed that in the intestinal microbiota of the Illness *Giardia* sp., Illness *Eimeria lamae*, *Giardia* sp. Negative, and *Eimeria lamae* negative groups, the classes Stramenopiles (12.16%, 15.98%, 28%, and 43.61%), Alveolata (45.3%, 13.6%, 12.65%, and 28%), and Rhizaria (26.6%, 51.9%, 17.3%, and 3.5%) accounted for 90% of the protist composition in the alpaca microbiota.

### 3.5. Detection of Modifications in the Taxonomic Composition Linked to the Influence of Parasites

Linear Discriminant Analysis (LDA) was utilized to assess effect size and delineate differences in the bacterial composition between sick alpacas infected with *Giardia* sp. and *Eimeria lamae*, and *Giardia* sp. negative and *Eimeria lamae* negative groups.

**Figure 5:**
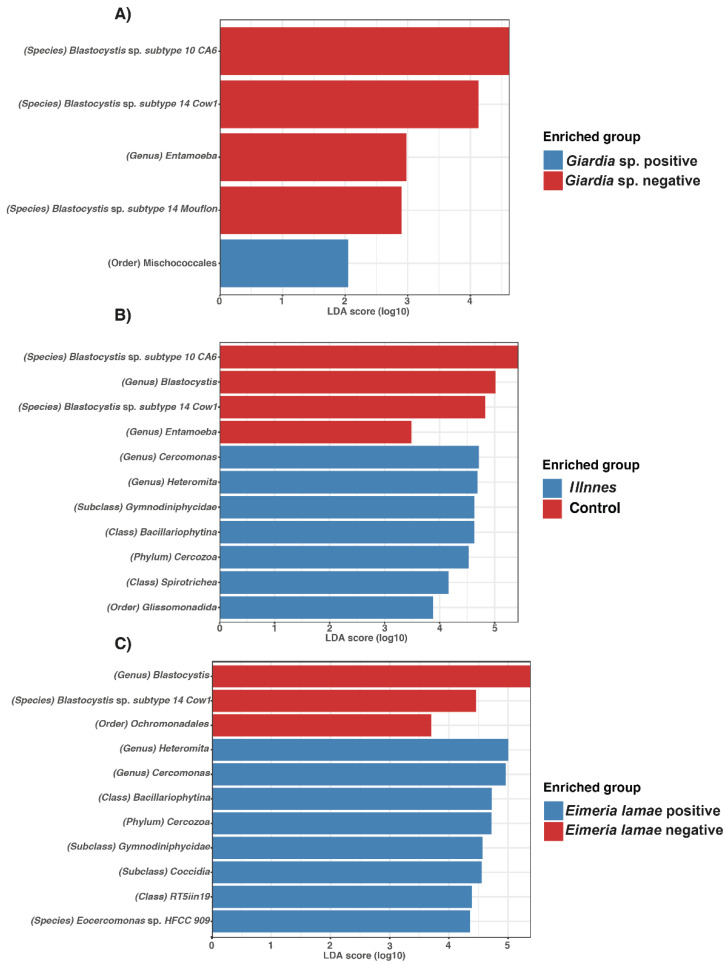
Differences in the intestinal protist microbiota among various health states in alpacas. Bar chart of Linear Discriminant Analysis (LDA) for differentially abundant genera. (**A**) Alpacas positives with *Giardia* sp. versus *Giardia* sp. negative group. (**B**) All unhealthy alpacas versus negative (control). (**C**) Alpacas positives with *Eimeria lamae* versus *Eimeria lamae* negative group.

In Figure 5, protists were identified. In the *Giardia* sp.-infected group, the presence of Mischococcales was observed (Figure 5A). Furthermore, genera such as *Cercomonas*, *Heteromita*, Gymnodiniphyicidae, Bacillariophytina, Cercozoa, Spirotrichea, and Glissomonadida were identified in sick alpacas (Figure 5B). Finally, the *Eimeria lamae*-infected group exhibited the presence of *Heteromita*, *Cercomonas*, Bacillariophytina, Cercozoa, Gymnodiniphyicidae, Coccidia, RT5iin19, and *Eocercomonas* sp. HFCC 909 (Figure 5C).

In comparison to the *Giardia* sp. negative group, various taxa were identified, including *Blastocystis* sp. Subtype 10 CA6, *Blastocystis* sp. Subtype 14 Cow1, *Entamoeba* and *Blastocystis* sp. Subtype 14 Mouflon (Figure 5A). Additionally, the control group showed the presence of *Blastocystis* sp. subtype 10 CA6, *Blastocystis*, *Blastocystis* sp. subtype 14 Cow1, and *Entamoeba* (Figure 5B). The *Eimeria lamae* negative group displayed *Blastocystis*, *Blastocystis* sp. subtype 14 Cow1, and Ochromonadales (Figure 5C).

**Figure 6:**
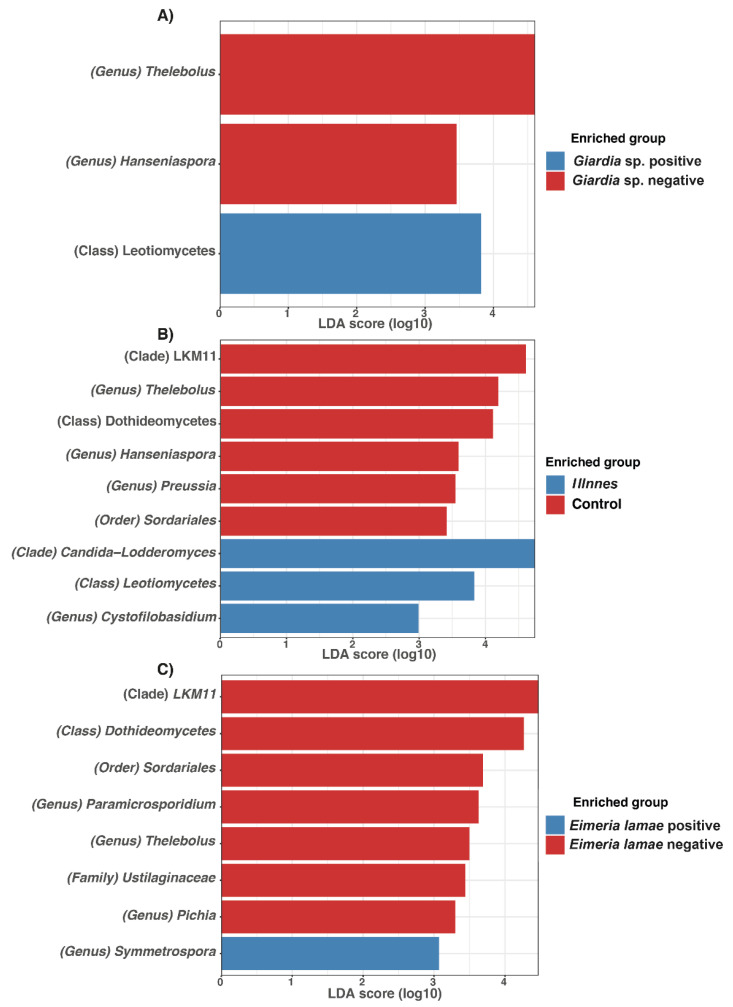
Differences in the intestinal fungal microbiota among different health states in alpacas. Linear Discriminant Analysis (LDA) bar chart displaying differentially abundant genera. (**A**) Alpacas positives with *Giardia* sp. versus *Giardia* sp. negative group. (**B**) All unhealthy alpacas versus negative (control). (**C**) Alpacas positives with *Eimeria lamae* versus *Eimeria lamae* negative group.

In Figure 6, fungi were identified. In the group infected with *Giardia* sp., the presence of Leotiomycetes was observed (Figure 6A). Additionally, in sick alpacas, genera such as Candida-Lodderomyces, Leotiomycetes, and *Cystofilobasidium* were identified (Figure 6B). Finally, the *Eimeria lamae*-infected group exhibited the presence of *Symmetrospora* (Figure 6C).

In comparison to the *Giardia* sp. group, Thelebolus and Hanseniaspora were identified (Figure 6A). Additionally, the control group displayed the presence of LKM11, *Thelebolus*, Dothideomycetes, *Hanseniaspora*, *Preussia*, and Sordariales (Figure 6B). *Eimeria lamae* negative group showed LKM11, Dothideomycetes, Sordariales, *Paramicrosporidium*, *Thelebolus*, Ustilaginaceae, and *Pichia* (Figure 6C).

## 4. Discussion

This study conducts the first analysis of fungi and protists in the intestinal microbiota of alpacas, comparing those affected by *Eimeria lamae* and *Giardia* sp. with healthy counterparts, filling a gap in existing research. Fungi and protists coexist and interact with other microorganisms in the intestinal tract of mammals [40]. Additionally, certain protozoa belonging to the protist group are recognized as pathogens, and their presence can influence the modulation of the intestinal microbiota composition in ruminants [41] and other mammals [42].

In the analysis of alpha diversity of ASVs corresponding to protist communities, significant differences were identified, with higher richness observed in sick alpacas with *Giardia* sp. and *Eimeria lamae* (Figure 1A and Figure 2A). On the other hand, in fungal communities (Figure 1B and Figure 2B), richness remained constant. In the context of a clinical presentation of diarrhea, a significant imbalance in the composition of the intestinal microbiota becomes evident, leading to an increase in opportunistic microorganisms [43]. These opportunistic microorganisms are predominantly parasitic in nature [44]. A comprehensive study has revealed a pronounced richness of protozoan parasites in the intestinal microbiota of mammals affected by this pathological condition [42,45]. Our finding unequivocally demonstrates that in the context of a condition such as diarrhea, there is a significant increase in the population of protists, particularly protozoans, in individuals afflicted by this physiological disturbance. This increase in protists is noteworthy due to their role in modulating bacterial and fungal communities through predatory activity [46]. They play a pivotal role in regulating changes in the structure and dynamics of these microorganisms [47], thus exerting a substantial influence on the intestinal microbiota of alpacas.

The influence of parasites on the composition of the microbiota is significantly related to the health status, with no discernible differentiated effect attributable to the type of parasite. Research conducted with pandas has emphasized the pivotal role of protists in shaping the intestinal microbiota, highlighting their predatory activities [48]. Furthermore, a significant presence of protists has been documented in non-human primates [49]. In contrast, prior investigations have not assigned significant importance to eukaryotes in human individuals experiencing diarrhea as a result of *Clostridioides difficile* infection [43,50]. It is worth noting that variability in the composition of protists is associated with factors that can influence resource availability and interactions among protists and other microorganisms in the intestinal microbiome, thereby affecting the structure of the protist community [48]. In light of this, the need for further similar studies to assess the impact of parasites on the beta diversity of ruminants and other mammals is underscored.

A significant inverse correlation was observed between alpha diversity indices (Figure 3), specifically Shannon, Simpson, and Pielou indices, and the biochemical parameter hemoglobin. *Giardia* sp. does not directly impact hemoglobin levels [51], in the same way that *Eimeria lamae* does not affect hemoglobin levels [52]. However, both pathogens induce diarrheal conditions, diarrhea can lead to a series of perturbations in the immune system. These immune disruptions can lead to a cascade of effects, such as increased susceptibility to infections and reduced immune surveillance. Ultimately, this immune dysregulation translates into a complex biological response that directly affects hemoglobin levels [53].

Furthermore, considering the specific pathological manifestations associated with *Giardia* sp. and *Eimeria lamae* in alpacas expands upon the impact of these parasites on the health of the host. Lesions observed in the epithelium of the jejunum and ileum villi, such as eosinophil infiltration, hyperemia, and epithelial denudation, underline the pathological impact of these infections [54,55]. These specific manifestations signify a direct effect on the intestinal health of alpacas, potentially influencing the immune response and overall health, which might correlate with alterations in the microbiota, warranting further investigation.

The results obtained regarding the taxonomy of fungi in alpacas (Figure 4A) show a high degree of concordance with previous studies on ruminants [56,57,58,59,60,61]. Various studies have identified Ascomycota and Basidiomycota as the most predominant fungal phyla in mammals and ruminants, as seen in cows [56,57,58], buffaloes [59], and goats [60,61]. Similarly, the classes identified in alpaca protists in our study (Figure 4B) have been reported in previous research on mammals, including humans [43,50], pandas [48], primates [49], and cows [57]. However, there is limited research on the gut microbiota of fungi and protists in alpacas.

The presence of Coccidia has been observed In individuals affected by *Eimeria lamae* (Figure 5C) [16]. This parasite is commonly found in newborn alpacas, leading to severe gastrointestinal manifestations, including diarrhea in approximately 80% of cases, and, in more critical situations, the death of crias aged between 1 to 2 months [62]. The severity of the infection can be particularly pronounced in captive camelids. This is because, in wild environments, camelids less frequently exhibit noticeable clinical signs, possibly due to a greater capacity to excrete a more substantial number of oocysts in various environments, thereby reducing the likelihood of subsequent infection or a higher parasitic burden in the organism [63].

In control groups (Figure 5), irrespective of parasite, the presence of various *Blastocystis* subtypes was detected. While *Blastocystis* is known to potentially induce gastrointestinal symptoms, such as diarrhea, constipation, abdominal pain, and flatulence [64], it has also been reported in several studies to be present in alpacas without any clinical signs of disease [58,65]. Additionally, this parasite has been documented in captive wild animals [66,67]. Therefore, it is suggested that the colonization of *Blastocystis* may serve as a reasonable marker for the assessment of gastrointestinal health [60]. Similarly, the presence of the pathogen *Entamoeba* was observed in alpacas from the control group (Figure 5A,B). Notably, the presence of this pathogen has been reported in healthy alpacas [68] and in animals from zoological collections [69,70]. This parallels the situation with *Blastocystis*, where no clinical symptoms are evident in these healthy alpacas, despite the pathogen’s presence.

Genus *Preussia* was identified in the total control group of alpacas (Figure 6B), and the presence of Genus *Pichia* was also noted in the two-month-old control group of alpacas (Figure 6C). This occurrence has similarly been documented in yaks [71]. The presence of these fungal genera in healthy alpacas can be attributed to the strong antibacterial capacity of *Preussia* [72] and its antioxidative properties [73], which are heightened as they age. These characteristics contribute to their resilience against diseases, bolstering their immune system and enhancing their environmental adaptability. In the case of *Pichia*, it effectively inhibits *Candida* infection [74].

Therefore, this is the first study of alpacas infected with parasites *Eimeria lamae* and *Giardia* sp., assessing the changes in the fungal and protist intestinal microbiota. Hence, further in-depth research is required to fully comprehend the implications of these infections on alpaca health and microbiota balance, as well as their potential impact on the immune response and adaptation to various environments.

## 5. Conclusions

In this study, an investigation was conducted into the diversity and composition of intestinal fungi and protists in alpacas affected by *Eimeria lamae* and *Giardia* sp., with a comparison made with healthy alpacas. The results showed increased protist diversity and abundance in infected alpacas, while fungal richness remained largely consistent. Regarding the composition of the intestinal microbiota, it was observed that both fungi and protists exhibited variations influenced by their health status. This study provides valuable insights into the impact of these parasites on alpaca intestinal microbiota, emphasizing the need for further research to understand their effects on health and immune responses in alpacas. However, it is crucial to acknowledge certain limitations inherent in the study. Illumina sequencing may introduce bias in capturing microbial diversity, suggesting that future studies incorporating PacBio-HiFi sequencing could offer a more comprehensive assessment. Additionally, the inclusion of further biochemical variables in subsequent research would enrich our understanding of the intricate dynamics within the alpaca intestinal microbiota.

## Figures and Tables

**Table 1:** Two-way PERMANOVA of the Bray–Curtis distance between the age and health status of the alpacas.

Items	Df	SumOfSqs	R2	F	Pr (>F)
Protists					
Influenced by parasites	1	0.3052	0.04171	0.8475	0.7089
Health status	1	1.1938	0.16313	3.3146	0.0003 ***
Influenced by parasites: Health status	1	0.4165	0.05691	1.1564	0.2005
**Fungi**					
Influenced by parasites	1	0.17297	0.07116	1.537	0.11
Health status	1	0.27745	0.11414	2.4654	0.0092 **
Influenced by parasites: Health status	1	0.17985	0.07399	1.5982	0.0906

(**) and (***) implicit significance (*p* < 0.01 and *p* < 0.001, respectively).

## Data Availability

The raw data supporting the conclusions of this article will be made available by the authors on request.

## Supplementary Materials

The following supporting information can be downloaded at: https://www.mdpi.com/article/10.3390/life14020187/s1, Figure S1: species richness rarefaction curves show the sequencing depth of 18S data obtained from fungi from gut samples. Figure S2: species richness rarefaction curves show the sequencing depth of 18S data obtained from protists from gut samples. Table S1: ooquistes per gram of feces (OPG) in 2-month-old alpacas with and without diarrhea infected with Eimeria lamae. Table S2: biochemical parameters of alpacas about their health status. H: hemoglobin; L: leukocyte; HR: heart rate; BF: respiratory rate.

## References

[1] Rosadio A.R., Maturrano H.L., Pérez J.D., Luna E.L. (2012). El complejo entérico neonatal en alpacas andinas. Rev. Investig. Vet. Perú.

[2] Cordero F.A., Contreras P.J., Mayhua M.P., Jurado E.M., Castrejón V.M. (2011). Correlaciones Fenotípicas Entre Características Productivas En Alpacas Huacaya. Rev. Investig. Vet. Perú.

[3] Quispe E.C., Rodríguez T.C., Iñiguez L.R., Mueller J.P. (2009). Producción de Fibra de Alpaca, Llama, Vicuña y Guanaco En Sudamérica. Anim. Genet. Resour. Inf..

[4] Bustinza Choque A.V., Machaca Machaca V., Cano Fuentes V., Quispe Coaquira J. (2021). Evolución y Desarrollo de Las Razas de Alpaca: Suri y Huacaya. Rev. Investig. Vet. Perú.

[5] Whitehead C.E. (2009). Neonatal Diseases in Llamas and Alpacas. Vet. Clin. North Am. Food Anim. Pract..

[6] Rojas M., Manchego A., Rocha C.B., Fornells L.A., Silva R.C., Mendes G.S., Dias H.G., Sandoval N., Pezo D., Santos N. (2016). Outbreak of Diarrhea among Preweaning Alpacas (*Vicugna pacos*) in the Southern Peruvian Highland. J. Infect. Dev. Ctries..

[7] Heller M.C., Chigerwe M. (2018). Diagnosis and Treatment of Infectious Enteritis in Neonatal and Juvenile Ruminants. Vet. Clin. North Am. Food Anim. Pract..

[8] Cebra C.K., Mattson D.E., Baker R.J., Sonn R.J., Dearing P.L. (2003). Potential Pathogens in Feces from Unweaned Llamas and Alpacas with Diarrhea. J. Am. Vet. Med. Assoc..

[9] Fowler M.E. (1989). Medicine and Surgery of South American Camelids: Llama, Alpaca, Vicuña, Guanaco.

[10] Cheney J.M., Allen G.T. (1989). Parasitism in Llamas. Vet. Clin. North Am. Food Anim. Pract..

[11] Whitehead C.E., Anderson D.E. (2006). Neonatal Diarrhea in Llamas and Alpacas. Small Rumin. Res..

[12] Guerrero C.A. (1967). Coccidia (Protozoa: Eimeriidae) of the Alpaca *Lama pacos*. J. Protozool..

[13] Guerrero C.A., Hernández J., Bazalar H., Alva J. (1971). *Eimeria macusaniensis* n. sp. (Protozoa: Eimeriidae) of the Alpaca Lama Pacos. J. Protozool..

[14] Santin M. (2020). *Cryptosporidium* and *Giardia* in Ruminants. Vet. Clin. North Am. Food Anim. Pract..

[15] Kiorpes A.L., Kirkpatrick C.E., Bowman D.D. (1987). Isolation of *Giardia* from a Llama and from Sheep. Can. J. Vet. Res..

[16] Gomez-Puerta L.A., Lopez-Urbina M.T., Alarcon V., Cama V., Gonzalez A.E., Xiao L. (2014). Occurrence of *Giardia duodenalis* Assemblages in Alpacas in the Andean Region. Parasitol. Int..

[17] Gómez-Couso H., Ortega-Mora L.M., Aguado-Martínez A., Rosadio-Alcántara R., Maturrano-Hernández L., Luna-Espinoza L., Zanabria-Huisa V., Pedraza-Díaz S. (2012). Presence and Molecular Characterisation of *Giardia* and *Cryptosporidium* in Alpacas (*Vicugna pacos*) from Peru. Vet. Parasitol..

[18] Koehler A.V., Rashid M.H., Zhang Y., Vaughan J.L., Gasser R.B., Jabbar A. (2018). First Cross-Sectional, Molecular Epidemiological Survey of *Cryptosporidium*, *Giardia* and *Enterocytozoon* in Alpaca (*Vicugna pacos*) in Australia. Parasit. Vectors.

[19] Trout J.M., Santín M., Fayer R. (2008). Detection of Assemblage A, *Giardia duodenalis* and *Eimeria* spp. in Alpacas on Two Maryland Farms. Vet. Parasitol..

[20] Partida-Rodríguez O., Serrano-Vázquez A., Nieves-Ramírez M.E., Moran P., Rojas L., Portillo T., González E., Hernández E., Finlay B.B., Ximenez C. (2017). Human Intestinal Microbiota: Interaction between Parasites and the Host Immune Response. Arch. Med. Res..

[21] Zhou Z., Wang Z., Cao L., Hu S., Zhang Z., Qin B., Guo Z., Nie K. (2013). Upregulation of Chicken TLR4, TLR15 and MyD88 in Heterophils and Monocyte-Derived Macrophages Stimulated with *Eimeria tenella* In Vitro. Exp. Parasitol..

[22] Zhao Y., Zhang K., Zou M., Sun Y., Peng X. (2018). Gga-miR-451 Negatively Regulates *Mycoplasma gallisepticum* (HS Strain)-Induced Inflammatory Cytokine Production via Targeting YWHAZ. Int. J. Mol. Sci..

[23] Cotton J.A., Amat C.B., Buret A.G. (2015). Disruptions of Host Immunity and Inflammation by *Giardia duodenalis*: Potential Consequences for Co-Infections in the Gastro-Intestinal Tract. Pathogens.

[24] Kim H.B., Isaacson R.E. (2015). The Pig Gut Microbial Diversity: Understanding the Pig Gut Microbial Ecology through the next Generation High Throughput Sequencing. Vet. Microbiol..

[25] Hadziavdic K., Lekang K., Lanzen A., Jonassen I., Thompson E.M., Troedsson C. (2014). Characterization of the 18S rRNA Gene for Designing Universal Eukaryote Specific Primers. PLoS ONE.

[26] Delzenne N.M., Knudsen C., Beaumont M., Rodriguez J., Neyrinck A.M., Bindels L.B. (2019). Contribution of the Gut Microbiota to the Regulation of Host Metabolism and Energy Balance: A Focus on the Gut–Liver Axis. Proc. Nutr. Soc..

[27] Henderson G., Cox F., Ganesh S., Jonker A., Young W., Janssen P.H. (2015). Rumen Microbial Community Composition Varies with Diet and Host, but a Core Microbiome Is Found across a Wide Geographical Range. Sci. Rep..

[28] Bowman D.D. (2022). Georgi. Parasitología Para Veterinarios.

[29] Barriga O.O. (2002). Las Enfermedades Parasitarias de los Animales Domésticos en la América Latina.

[30] Cebra C.K., Stang B.V. (2008). Comparison of Methods to Detect Gastrointestinal Parasites in Llamas and Alpacas. J. Am. Vet. Med. Assoc..

[31] Vega L., Jaimes J., Morales D., Martínez D., Cruz-Saavedra L., Muñoz M., Ramírez J.D. (2021). Microbial Communities’ Characterization in Urban Recreational Surface Waters Using Next Generation Sequencing. Microb. Ecol..

[32] Bolyen E., Rideout J.R., Dillon M.R., Bokulich N.A., Abnet C.C., Al-Ghalith G.A., Alexander H., Alm E.J., Arumugam M., Asnicar F. (2019). Reproducible, Interactive, Scalable and Extensible Microbiome Data Science Using QIIME 2. Nat. Biotechnol..

[33] Callahan B.J., McMurdie P.J., Rosen M.J., Han A.W., Johnson A.J.A., Holmes S.P. (2016). DADA2: High-Resolution Sample Inference from Illumina Amplicon Data. Nat. Methods.

[34] Katoh K., Misawa K., Kuma K., Miyata T. (2002). MAFFT: A novel method for rapid multiple sequence alignment based on fast fourier transform. Nucleic Acids Res..

[35] McMurdie P.J., Holmes S. (2013). Phyloseq: An R package for reproducible interactive analysis and graphics of microbiome census data. PLoS ONE.

[36] R Core Team (2020). R: A Language and Environment for Statistical Computing.

[37] Xu S., Zhan L., Tang W., Wang Q., Dai Z., Zhou L., Feng T., Chen M., Wu T., Hu E. (2023). MicrobiotaProcess: A comprehensive R package for deep mining microbiome. Innovation.

[38] Anderson M.J. (2017). Permutational multivariate analysis of variance (PERMANOVA). Wiley StatsRef: Statistics Reference Online.

[39] Oksanen J., Blanchet F.G., Friendly M., Kindt R. (2022). Vegan: Community Ecology Package. R Package Version 2.6-4. http://cran.r-project.org/package=vegan.

[40] Richard M.L., Sokol H. (2019). The gut mycobiota: Insights into analysis, environmental interactions and role in gastrointestinal diseases. Nat. Rev. Gastroenterol. Hepatol..

[41] Cholewińska P., Czyż K., Nowakowski P., Wyrostek A. (2020). The microbiome of the digestive system of ruminants—A review. Anim. Health Res. Rev..

[42] Gilchrist C.A., Petri S.E., Schneider B.N., Reichman D.J., Jiang N., Begum S., Watanabe K., Jansen C.S., Elliott K.P., Burgess S.L. (2016). Role of the Gut Microbiota of Children in Diarrhea Due to the Protozoan Parasite *Entamoeba histolytica*. J. Infect. Dis..

[43] Herrera G., Vega L., Patarroyo M.A., Ramírez J.D., Muñoz M. (2021). Gut microbiota composition in health-care facility-and community-onset diarrheic patients with *Clostridioides difficile* Infection. Sci. Rep..

[44] Janeway C., Travers P., Walport M., Shlomchik M.J. (2001). Immunobiology: The Immune System in Health and Disease.

[45] Alzate J.F., Toro-Londoño M., Cabarcas F., Garcia-Montoya G., Galvan-Diaz A. (2020). Contrasting microbiota profiles observed in children carrying either *Blastocystis* spp. or the commensal amoebas *Entamoeba coli* or *Endolimax nana*. Sci. Rep..

[46] Pernthaler J. (2005). Predation on prokaryotes in the water column and its ecological implications. Nat. Rev. Microbiol..

[47] Johnke J., Cohen Y., de Leeuw M., Kushmaro A., Jurkevitch E., Chatzinotas A. (2014). Multiple micro-predators controlling bacterial communities in the environment. Curr. Opin. Biotechnol..

[48] Zhu D., Lu L., Zhang Z., Qi D., Zhang M., O’Connor P., Wei F., Zhu Y.-G. (2021). Insights into the roles of fungi and protist in the giant panda gut microbiome and antibiotic resistome. Environ. Int..

[49] Mann A.E., Mazel F., Lemay M.A., Morien E., Billy V., Kowalewski M., Di Fiore A., Link A., Goldberg T.L., Tecot S. (2020). Biodiversity of protists and nematodes in the wild nonhuman primate gut. ISME J..

[50] Vega L., Herrera G., Muñoz M., Patarroyo M.A., Maloney J.G., Santín M., Ramírez J.D. (2021). Gut microbiota profiles in diarrheic patients with co-occurrence of *Clostridioides difficile* and *Blastocystis*. PLoS ONE.

[51] Gil F.F., Ventura L.L.A., Fonseca J.F., Saniago H.C., Busatti H., Santos J.F.G., Gomes M.A. (2018). Hematological profile in natural progression of giardiasis: Kinetics of experimental infection in gerbils. J. Infect. Dev. Ctries..

[52] Rakhshandehroo E., Nazifi S., Razavi S.M., Ghane M., Alavi A.M. (2013). Caprine coccidiosis: The effects of induced infection on certain blood parameters. Vet. Fak. Sveučilišta U Zagrebu.

[53] Ruiz A., Matos L., Muñoz M.C., Hermosilla C., Molina J.M., Andrada M., Rodríguez F., Pérez D., López A., Guedes A. (2013). Isolation of an *Eimeria ninakohlyakimovae* field strain (Canary Islands) and analysis of its infection characteristics in goat kids. Res. Vet. Sci..

[54] Cebra C. (2014). Disorders of the Digestive System. Llama Alpaca Care.

[55] Palacios E.C., Tabacchi N.L., Chavera C.A., López U.T., Santillán A.G., Sandoval C.N., Pezo C.D., Perales C.R. (2004). Eimeriosis En Crías de Alpacas: Estudio Anátomo Histopatológico. Rev. Investig. Vet. Perú.

[56] Azad E., Fehr K.B., Derakhshani H., Forster R., Acharya S., Khafipour E., McGeough E., McAllister T.A. (2020). Interrelationships of fiber-associated anaerobic fungi and bacterial communities in the rumen of bloated cattle grazing alfalfa. Microorganisms.

[57] Ramírez A.L., Herrera G., Muñoz M., Vega L., Cruz-Saavedra L., García-Corredor D., Pulido-Medellín M., Bulla-Castañeda D.M., Giraldo J.C., Bernal M.C. (2021). Describing the intestinal microbiota of Holstein *Fasciola*-positive and -negative cattle from a hyperendemic area of fascioliasis in central Colombia. PLoS Negl. Trop. Dis..

[58] Wang H., Li P., Liu X., Zhang C., Lu Q., Xi D., Yang R., Wang S., Bai W., Yang Z. (2019). The Composition of Fungal Communities in the Rumen of Gayals (*Bos frontalis*), Yaks (*Bos grunniens*), and Yunnan and Tibetan Yellow Cattle (*Bos taurs*). Pol. J. Microbiol..

[59] Chen X., An M., Zhang W., Li K., Kulyar M.F.-E.-A., Duan K., Zhou H., Wu Y., Wan X., Li J. (2022). Integrated Bacteria-Fungi Diversity Analysis Reveals the Gut Microbial Changes in Buffalo with Mastitis. Front. Vet. Sci..

[60] Lv Q.-B., Meng J.-X., Ma H., Liu R., Qin Y., Qin Y.-F., Geng H.-L., Ni H.-B., Zhang X.-X. (2023). Description of Gut Mycobiota Composition and Diversity of Caprinae Animals. Microbiol. Spectr..

[61] Fliegerova K.O., Podmirseg S.M., Vinzelj J., Grilli D.J., Kvasnová S., Schierová D., Sechovcová H., Mrázek J., Siddi G., Arenas G.N. (2021). The effect of a high-grain diet on the rumen microbiome of goats with a special focus on anaerobic fungi. Microorganisms.

[62] Sánchez-Herencia D., Mamani-Mango G., Coila-Añasco P. (2021). *Eimeria* control in baby alpacas using Toltrazuril as a prophylactic measure in humid Puna. J. Selva Andina Anim. Sci..

[63] Dubey J.P. (2018). A review of Coccidiosis in South American camelids. Parasitol. Res..

[64] Casero R.D., Mongi F., Sánchez A., Ramírez J.D. (2015). *Blastocystis* and urticaria: Examination of subtypes and morphotypes in an unusual clinical manifestation. Acta Trop..

[65] Ma Y., Wang C., Elmhadi M., Zhang H., Han Y., Shen B., He B.L., Liu X.Y., Wang H.R. (2021). Thiamine ameliorates metabolic disorders induced by a long-term high-concentrate diet and promotes rumen epithelial development in goats. J. Dairy Sci..

[66] Zhao G.H., Hu X.F., Liu T.L., Hu R.S., Yu Z.Q., Yang W.B., Wu Y.L., Yu S.K., Song J.K. (2017). Molecular Characterization of *Blastocystis* sp. in captive wild animals in Qinling Mountains. Parasitol. Res..

[67] Deng L., Yao J., Chen S., He T., Chai Y., Zhou Z., Shi X., Liu H., Zhong Z., Fu H. (2021). First identification and molecular subtyping of *Blastocystis* sp. in zoo animals in southwestern China. Parasit. Vectors.

[68] Gao W.-W., Ma Y.-T., Ma Y.-Y., Li R.-L., Li J., Zheng F.-G., Zheng W.-B., Liu Q., Zhu X.-Q. (2021). First report of *Eimeria* and *Entamoeba* infection in alpacas (*Vicugna pacos*) in Shanxi Province, northern China. Parasitol. Res..

[69] Qi T., Zheng W., Guo L., Sun Y., Li J., Kang M. (2023). First description of *Blastocystis* sp. and *Entamoeba* sp. infecting zoo animals in the Qinghai-Tibetan plateau area, China. Front. Cell. Infect. Microbiol..

[70] Liu J., Wang X., Zhang W., Kulyar M.F.-A., Ullah K., Han Z., Qin J., Bi C., Wang Y., Li K. (2022). Comparative analysis of gut microbiota in healthy and diarrheic yaks. Microb. Cell Factories.

[71] Li Y., Li X., Liu Y., Nie C., Chen C., Niu J., Zhang W. (2022). Comparison of bacterial and fungal community structure and potential function analysis of Yak feces before and after weaning. BioMed Res. Int..

[72] Mapperson R.R., Kotiw M., Davis R.A., Dearnaley J.D.W. (2014). The diversity and antimicrobial activity of *Preussia* sp. endophytes isolated from australian dry rainforests. Curr. Microbiol..

[73] Paudel B., Bhattarai K., Bhattarai H.D. (2018). Antimicrobial and antioxidant activities of two polyketides from lichen-endophytic fungus *Preussia* sp.. Z. Naturforschung C J. Biosci..

[74] Macauley-Patrick S., Fazenda M.L., McNeil B., Harvey L.M. (2005). Heterologous protein production using the *Pichia pastoris* expression system. Yeast Chichester Engl..

