# Supplementary material for: Parasitism-Induced Changes in Microbial Eukaryotes of Peruvian Alpaca Gastrointestinal Tract
Source: Life (Basel). 2024 Jan 27;14(2):187. doi: 10.3390/life14020187 (PMC10890412; doi:10.3390/life14020187)
Supplement: Supplementary file 1 [file life-14-00187-s001.zip › life-2738554-supplementary.pdf]

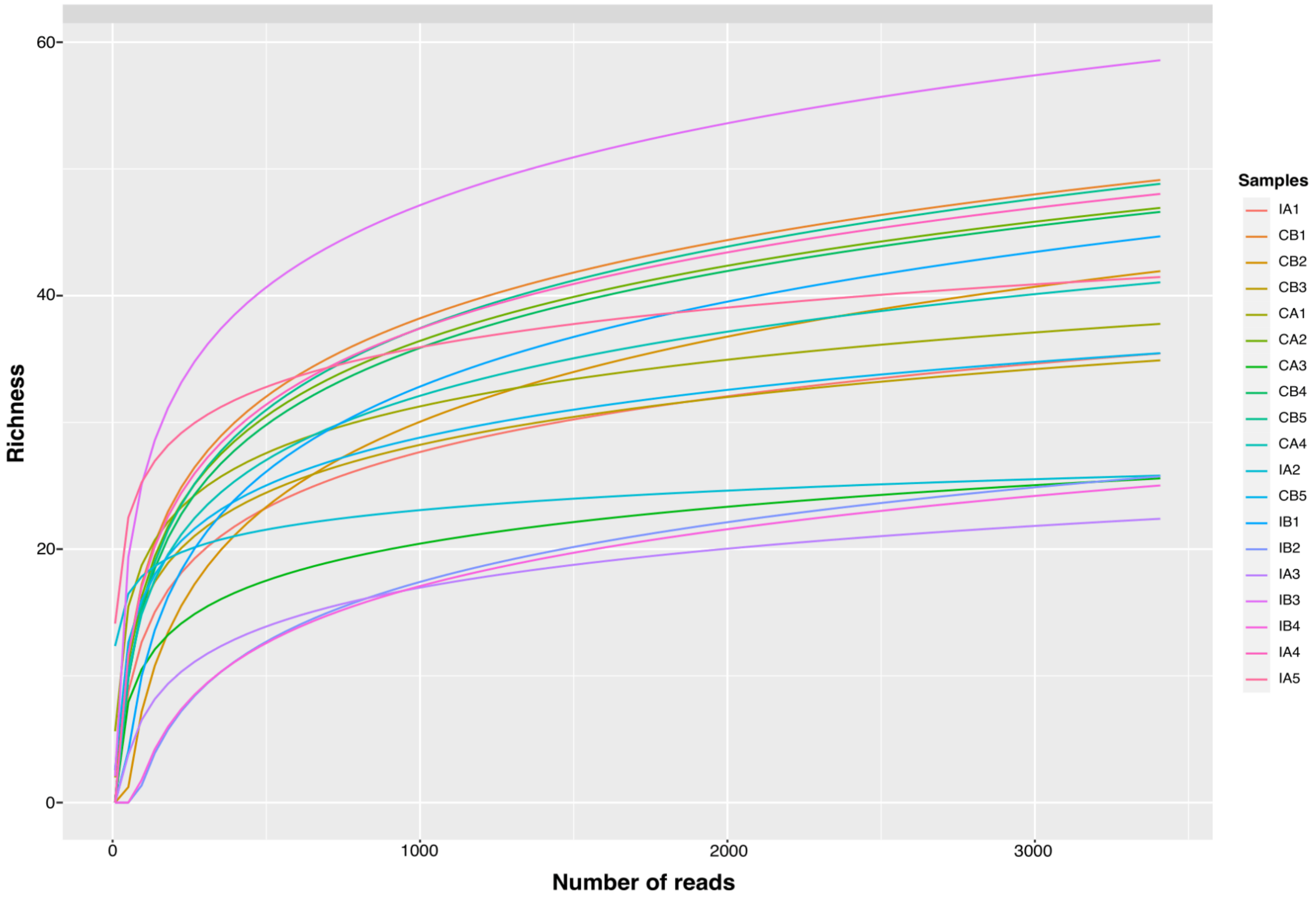

Figure S1. Species richness rarefaction curves show sequencing depth of 18S data obtained from fungi from gut samples.

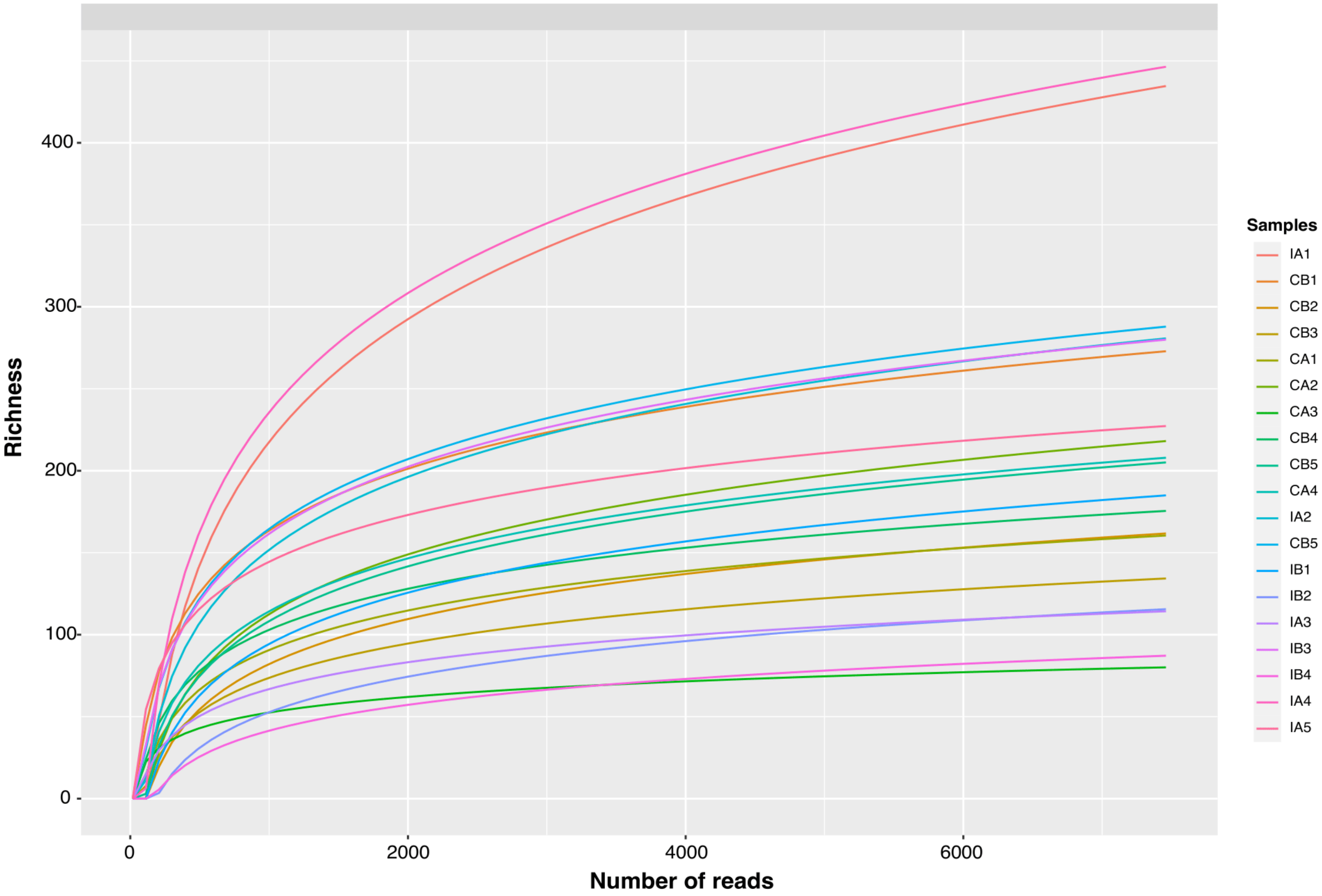

Figure S2. Species richness rarefaction curves show sequencing depth of 18S data obtained from protist from gut samples.

Table S1. Ooquistes per gram of feces (OPG) in 2-month-old alpacas with and without diarrhea infected with *Eimeria lamae*.

Age	Heatlh Condition	ID	Parasite	Ooquistes per gram of feces (OPG)
2 months	with diarrea	IB1	*E. lamae*	15750
		IB2	*E. lamae*	22450
		IB3	*E. lamae*	34800
		IB4	*E. lamae*	12750
	without diarrhea	CB1	*E. lamae*	0
		CB2	*E. lamae*	0
		CB3	*E. lamae*	0
		CB4	*E. lamae*	0
		CB5	*E. lamae*	0
		CB6	*E. lamae*	0

Table S2. Biochemical parameters of alpacas in relation to their health status. H: Hemoglobin; L: Leukocyte; HR: Heart Rate; BF: Respiratory Rate.

Samples	Health Condition	H (g/dL)	L (mm^3)	HR (bpm)	BF (bpm)
IA1	*Giardia sp.* positive	34.69	7950	172	42
IA2	*Giardia sp.* positive	36.36	9150	96	34
IA3	*Giardia sp.* positive	38.46	9425	138	48
IA4	*Giardia sp.* positive	38.46	6000	116	28
IA5	*Giardia sp.* positive	37.25	11850	96	38
IB1	*Eimeria lamae* positive	31.63	11500	110	28
IB2	*Eimeria lamae* positive	34	8000	126	36
IB3	*Eimeria lamae* positive	32.71	9200	134	34
IB4	*Eimeria lamae* positive	38.46	22100	116	34
CA1	*Giardia sp.* negative	30	6350	152	48
CA2	*Giardia sp.* negative	31.58	8750	128	34
CA3	*Giardia sp.* negative	30	8500	132	28
CA4	*Giardia sp.* negative	34.29	7850	100	26
CB1	*Eimeria lamae* negative	34.69	7950	172	42
CB2	*Eimeria lamae* negative	33.33	18400	148	26
CB3	*Eimeria lamae* negative	35	1750	148	40
CB4	*Eimeria lamae* negative	35.85	12800	130	15
CB5	*Eimeria lamae* negative	31	1550	142	26
CB6	*Eimeria lamae* negative	39.22	9550	188	44
